# Publisher Correction: HIF-1 stabilization in T cells hampers the control of *Mycobacterium tuberculosis* infection

**DOI:** 10.1038/s41467-022-33971-w

**Published:** 2022-11-01

**Authors:** Ruining Liu, Victoria Muliadi, Wenjun Mou, Hanxiong Li, Juan Yuan, Johan Holmberg, Benedict J. Chambers, Nadeem Ullah, Jakob Wurth, Mohammad Alzrigat, Susanne Schlisio, Berit Carow, Lars Gunnar Larsson, Martin E. Rottenberg

**Affiliations:** 1 Department of Microbiology, Tumor and Cell Biology, Karolinska Institutet, Stockholm, Sweden; 2 Department of Cell and Molecular Biology, Karolinska Institutet, Stockholm, Sweden; 3 Center for Infectious Medicine, Department of Medicine Huddinge, Karolinska Institutet, Stockholm, Sweden; 4 Present Address: Capital Children’s Hospital, Beijing, China; 5 Present Address: University Children’s Hospital Zurich, Zurich, Switzerland; 6 Present Address: Novovavax AB, Uppsala, Sweden

**Keywords:** Tuberculosis, T-helper 1 cells, Lymphocyte activation

Correction to: *Nature Communications* 10.1038/s41467-022-32639-9, published online 05 September 2022

The original version of this Article contained errors in Fig. 1 and Fig. 2. In the original version of Fig. 1, several labels in figure panels were illegible. The correct version of Fig. 1 is:
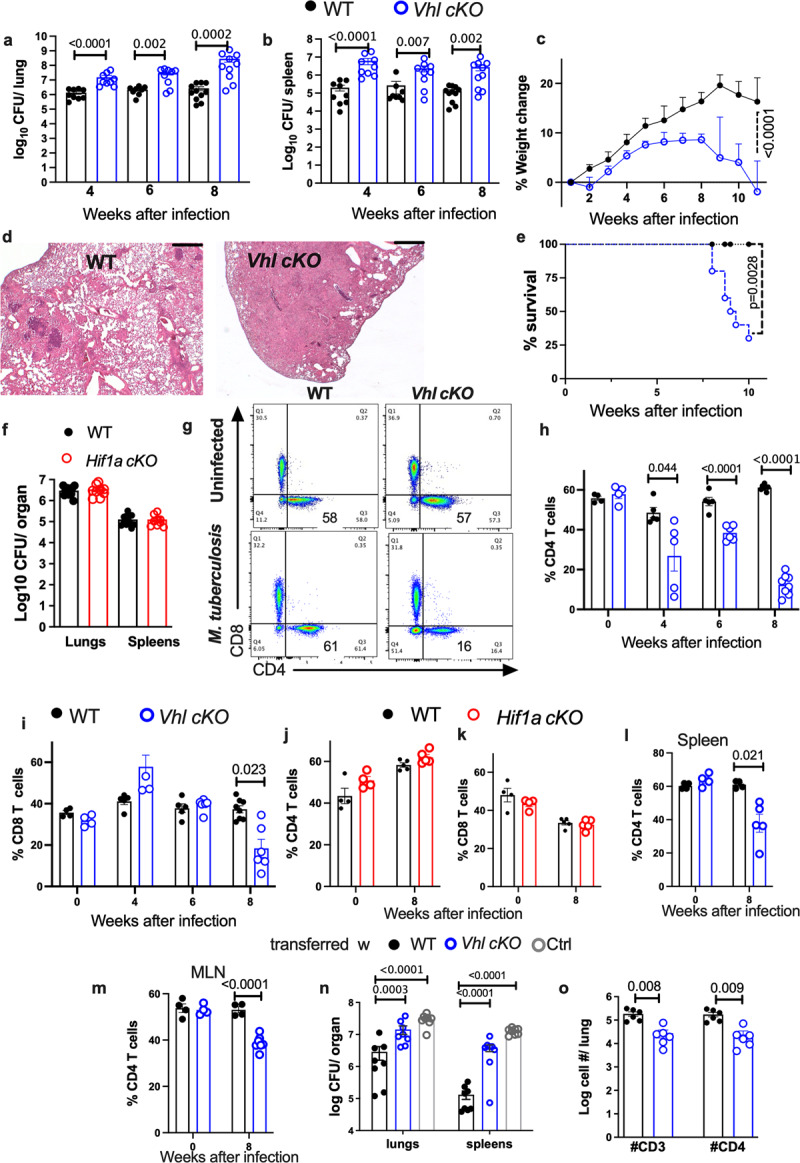


which replaces the previous incorrect version:
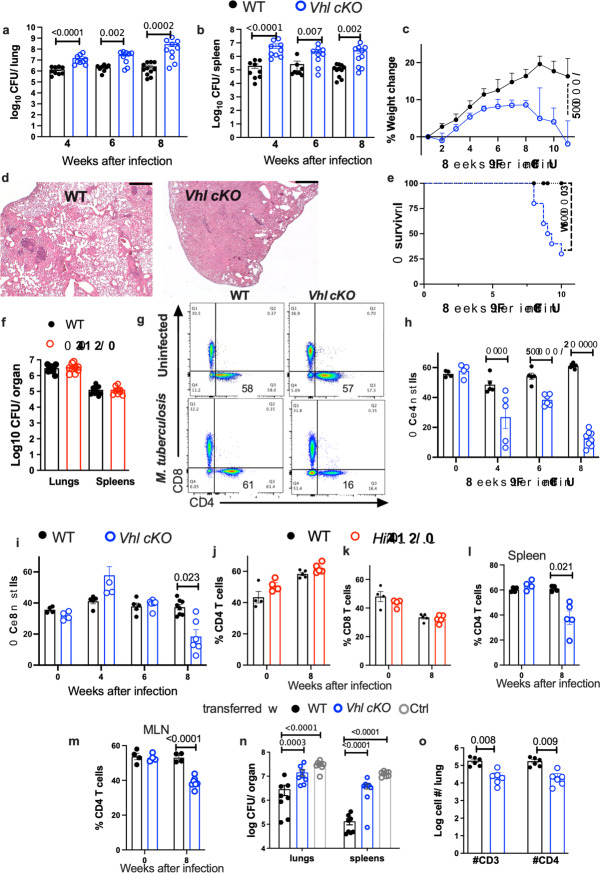


In the original version of Fig. 2, several labels in figure panels were illegible. The correct version of Fig. 2 is:
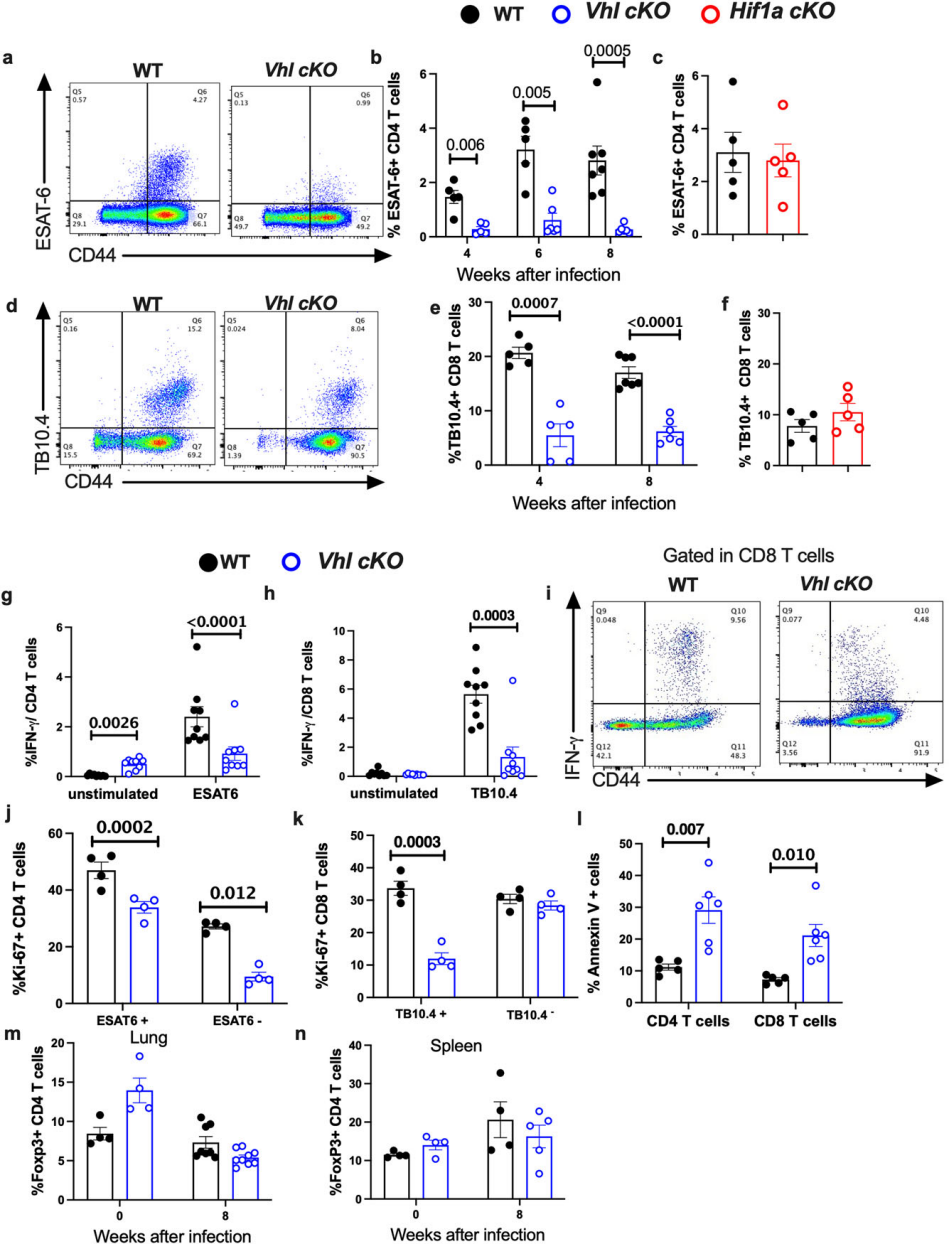


which replaces the previous incorrect version:
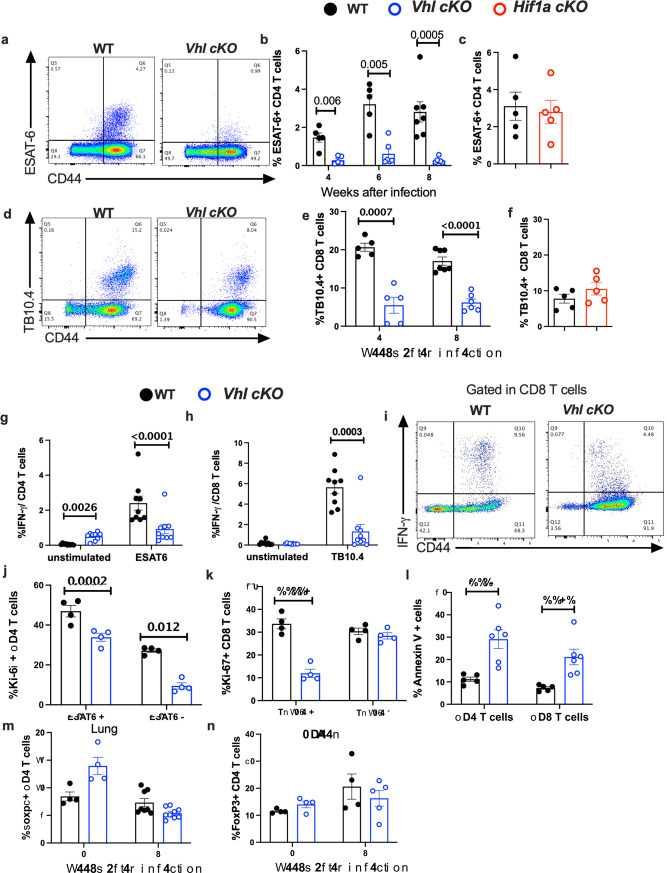


This has been corrected in both the PDF and HTML versions of the Article.

