# Supplementary material for: HIF-1 stabilization in T cells hampers the control of Mycobacterium tuberculosis infection
Source: Nat Commun. 2022 Sep 5;13:5093. doi: 10.1038/s41467-022-32639-9 (PMC9445005; doi:10.1038/s41467-022-32639-9)
Supplement: Supplementary file 1 — Supplementary Information [file 41467_2022_32639_MOESM1_ESM.pdf]

HIF-1 stabilization in T cells hampers the control of infection with *M. tuberculosis* by impairing T cell activation

By

Ruining Liu, Victoria Muliadi, Wenjun Mou, Hanxiong Li, Juan Yuan, Johan Holmberg, Benedict J. Chambers, Nadeem Ullah, Jakob Wurth, Mohammad Alzrigat, Susanne Schlisio, Berit Carow, Lars Gunnar Larsson and Martin E. Rottenberg

Supplementary Information

Supplementary figures 1-10

Supplementary tables 1-3

Supplementary figure 1

VHL expression in T cells is critical for the control of *M. tuberculosis*-infection in mice

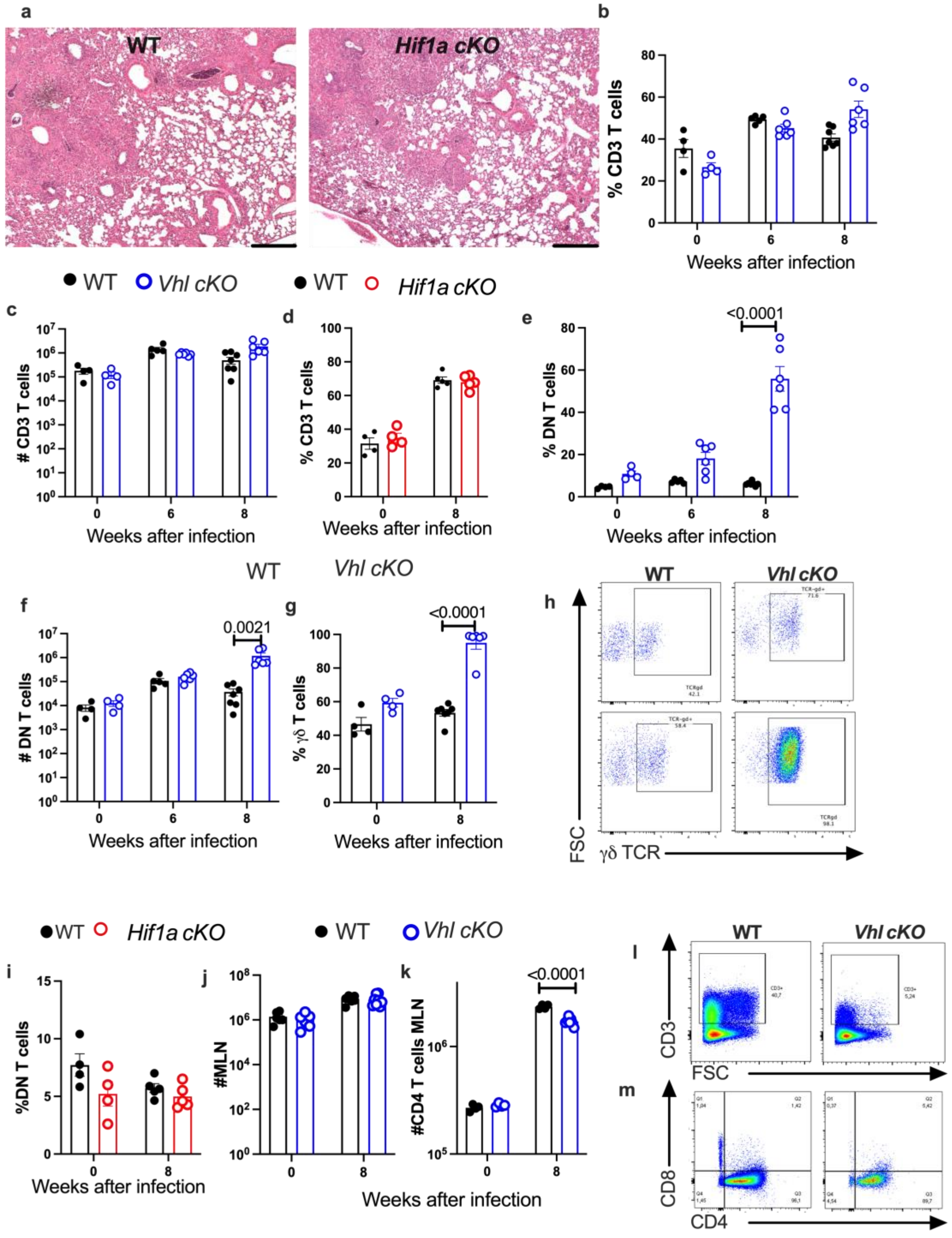

(a) Representative micrographs from hematoxylin-eosin-stained paraffin lung sections from *Vhl cKO* and WT mice 8 weeks after infection with *M. tuberculosis* (bar: 300 µm). (b, c) The frequency (b) and numbers (c) of CD3 T cells in the lungs of *Vhl cKO* and WT mice before and after *M. tuberculosis* infection. (d) The frequency of CD3 T cells in lungs from *Hif1a cKO* and WT mice before and after *M. tuberculosis* infection. (e-h) The frequency and numbers of DN (e, f) and γδ (g) (within DN T cells) and representative dot plots of γδ T cells within DN T cells (h) are shown. (i) The fraction of DN T cells in the lungs of *M. tuberculosis*-infected *Hif1a cKO* and WT mice is depicted. (j, k) The numbers of MLN (j) and of MLN CD4 T cells from WT and *Vhl cKO* mice before and after infection are depicted. (l, m) A representative dot plot of CD3 (l) and CD4 and CD8 (m) expression within T cells in lungs from *Rag2*$^{-/-}$ mice transferred with either WT or *Vhl cKO* CD4 T cells were determined 4 weeks after infection. Each symbol represents one mouse, and the data are presented as the mean ± SEM. (b, c, e, f, g) WT n=4, 5, 7; *Vhl cKO* n=4, 6, 6 mice at 0, 6 and 8 w.p.i. respectively; (d, i) WT n=4, 5; *Hif1a cKO* n=4, 5 mice at 0 and 8 w.p.i. respectively); (j) n=6 and 8 per group at 0 and 8 w.p.i; (k) WT n=4, 4 and *Vhl cKO* n=4, 7 at 0 and 8 w.p.i. respectively. The p values were calculated using a two-tailed unpaired *t* test with Welch's correction and FDR adjustment for multiple comparisons. Source data are provided as a Source Data file.

Supplementary figure 2

Increased T_{CM} in *Vhl cKO* mice infected with *M. tuberculosis*

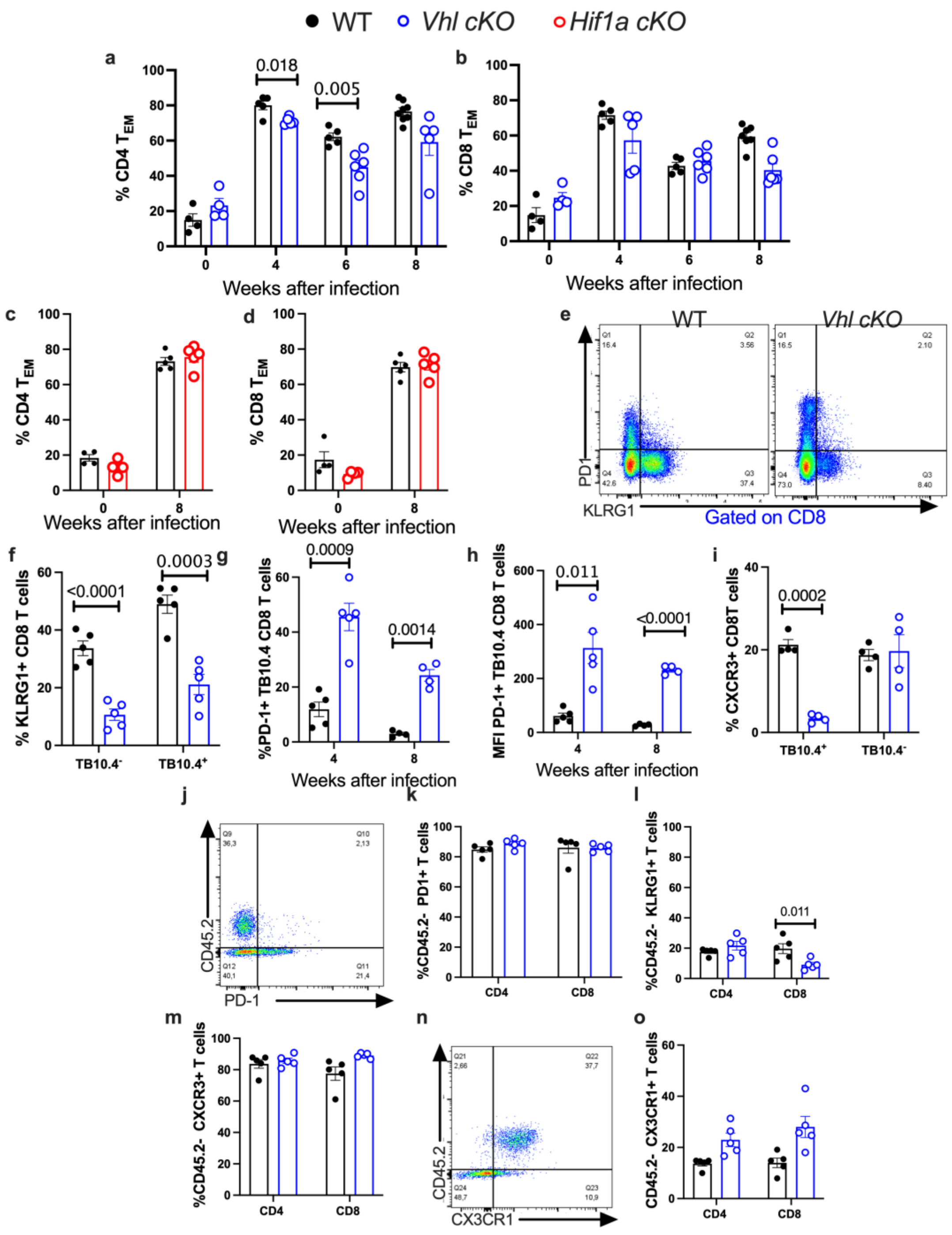

(a-d) The frequencies of CD4 (a) and CD8 (b) T_{EM} in the lung of *Vhl cKO* (a, b) *Hif1a cKO* (c, d) and WT mice before and after *M. tuberculosis* infection are shown. (e) Representative dot plot of PD-1 and KLRG1 expression on CD8 T cells from the lung of WT and *Vhl cKO* mice 8 weeks after infection with *M. tuberculosis*. (f) The frequency of KLRG1+ TB10.4-binding or not CD8 T cells in the lungs of *M. tuberculosis*-infected *Vhl cKO* and WT mice. (g, h) The frequency and MFI of PD-1+ TB10.4 tetramer binding CD8 T cells in the lungs of *M. tuberculosis*-infected *Vhl cKO* and WT mice is depicted. (i) The frequencies of CXCR3+ tetramer TB10.4 binding or not CD8 T cells in the lungs of *M. tuberculosis*-infected *Vhl cKO* and WT mice are depicted. (j-o) Dot plot of i.v. labelled PD-1+ (j) and CX3CR1 (n) CD44+CD4T cells in the lung of *Vhl cKO* mice 7 weeks after infection with *M. tuberculosis*. The percentage of CD45.2 negative (parenchymal) PD-1+ (k), KLRG1+ (l), CXCR3+ (m) and CX3CR1 (o) T cells in the lungs of *Vhl cKO* and WT mice 7 weeks after the infection with *M. tuberculosis*. Each symbol represents one mouse, and the data are presented as the mean ± SEM. (a, b) WT n=4, 5, 5, 8; *Vhl cKO* n=4, 5, 6 mice at 0, 4, 6 and 8 w.p.i. respectively; (c, d) n=4, 5 mice per group at 0 and 8 w.p.i. respectively; (f-h, k-m, o) n=5 mice per group; (i) n= 4 mice per group. The p values were calculated using a two-tailed unpaired *t* test with Welch's correction and FDR adjustment for multiple comparisons. Source data are provided as a Source Data file.

Supplementary Figure 3

VHL expression in lung CD4 T cells from *M. tuberculosis*-infected mice controls proliferation and effector responses

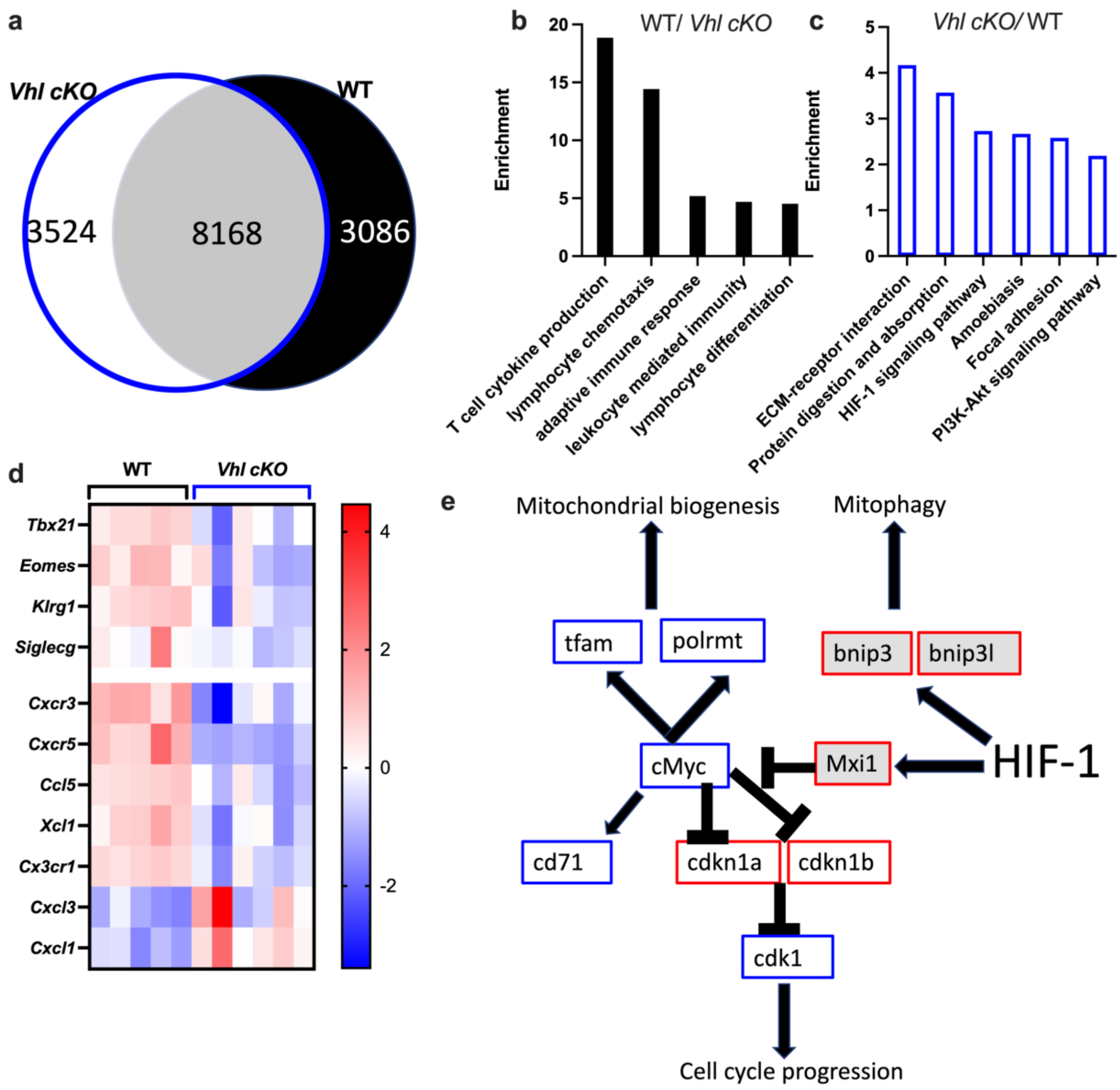

(a) The Venn diagram represents the number of genes determine by RNA seq that are common to or uniquely expressed within WT and *Vhl cKO* CD4 T cells from *M. tuberculosis* infected mice. (b,c) The enrichment ratio of the most significant terms after KEEG pathway

analysis comparing WT vs *Vhl cKO* CD4 T cells (b) and *Vhl cKO* vs WT (c) CD4 T cells is shown. (d) The heat maps of RNA-seq data showing selected, differentially expressed genes involved in responses to chemokines and T cell activation are shown in samples of WT and *Vhl cKO* CD4 T cells from *M. tuberculosis*-infected mice. (e) HIF-1-mediated proliferative and mitochondrial alterations suggested by the RNAseq data (in red upregulation and in blue downregulation in *Vhl cKO* CD4 T cells from *M. tuberculosis* infected mice). HIF-mediates induction of the MYC-antagonist *mxi1* coding gene [15, 34], hampering proliferation via de-repression of *Cdkn1a* and *Cdkn1b* and thereby inhibition of *cdk1*. Mitochondrial biogenesis is inhibited by reducing *Polrmt* and *Tfam* transcripts. HIF-mediated activation of mitophagy is suggested by the increase of *Bnip3* and *Bnip3l* mRNA.

Supplementary Figure 4

VHL expression is required for TCR activation of CD4 T cells

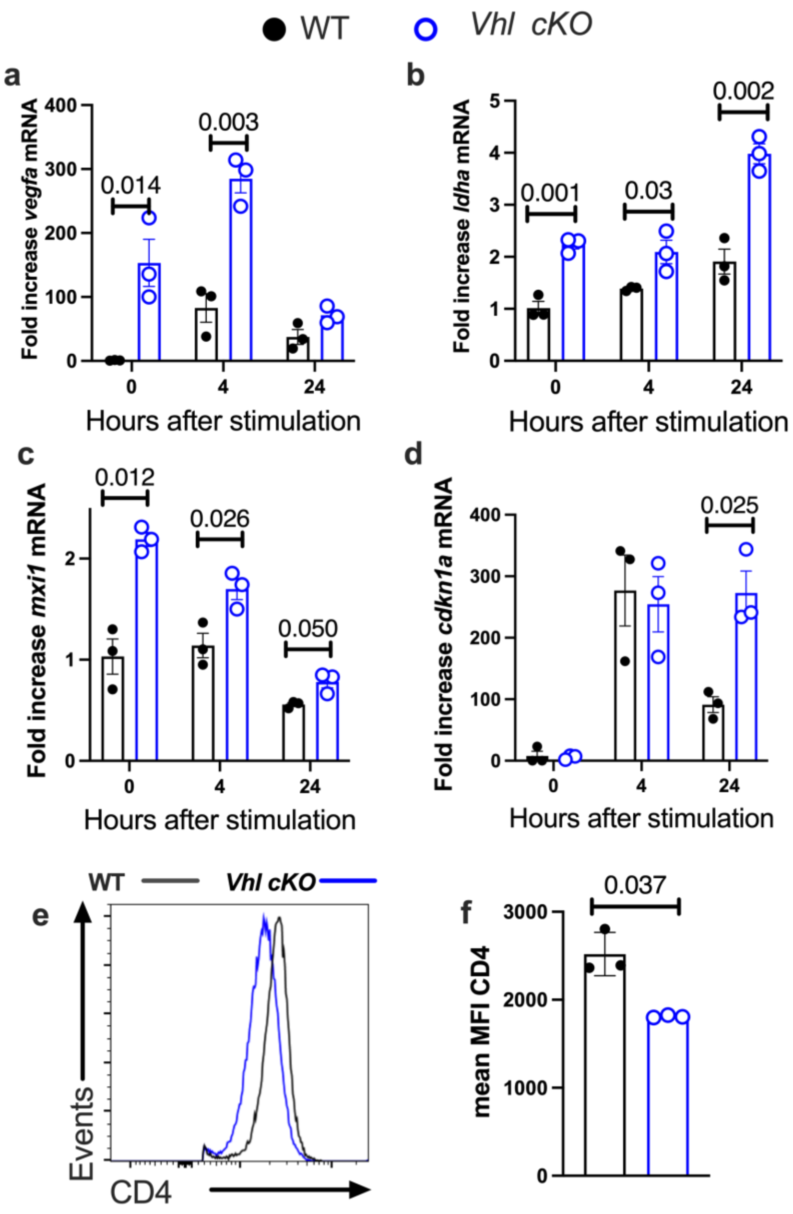

(a-d) *Vegf* (a), *Ldha* (b), *Mxi1* (c) and *Cdkn1a* (d) mRNA titers were measured by real time PCR in total RNA from *Vhl cKO* and WT CD4 T cells after anti-CD3/CD28 stimulation. The

fold increase of transcript levels (n=3 independent cultures per group) are depicted. (e, f) Representative histogram (e) and the MFI (f) of the CD4 expression in *Vhl cKO* and WT CD4 T cells (n=3 per group) are shown. Each symbol represents one mouse, and the data are presented as the mean ± SEM. The p-values were calculated using a two-tailed unpaired *t* test with Welch's correction and FDR adjustment for multiple comparisons. Source data are provided as a Source Data file.

Supplementary Figure 5

VHL promotes proliferation of TCR activated CD4 T cells

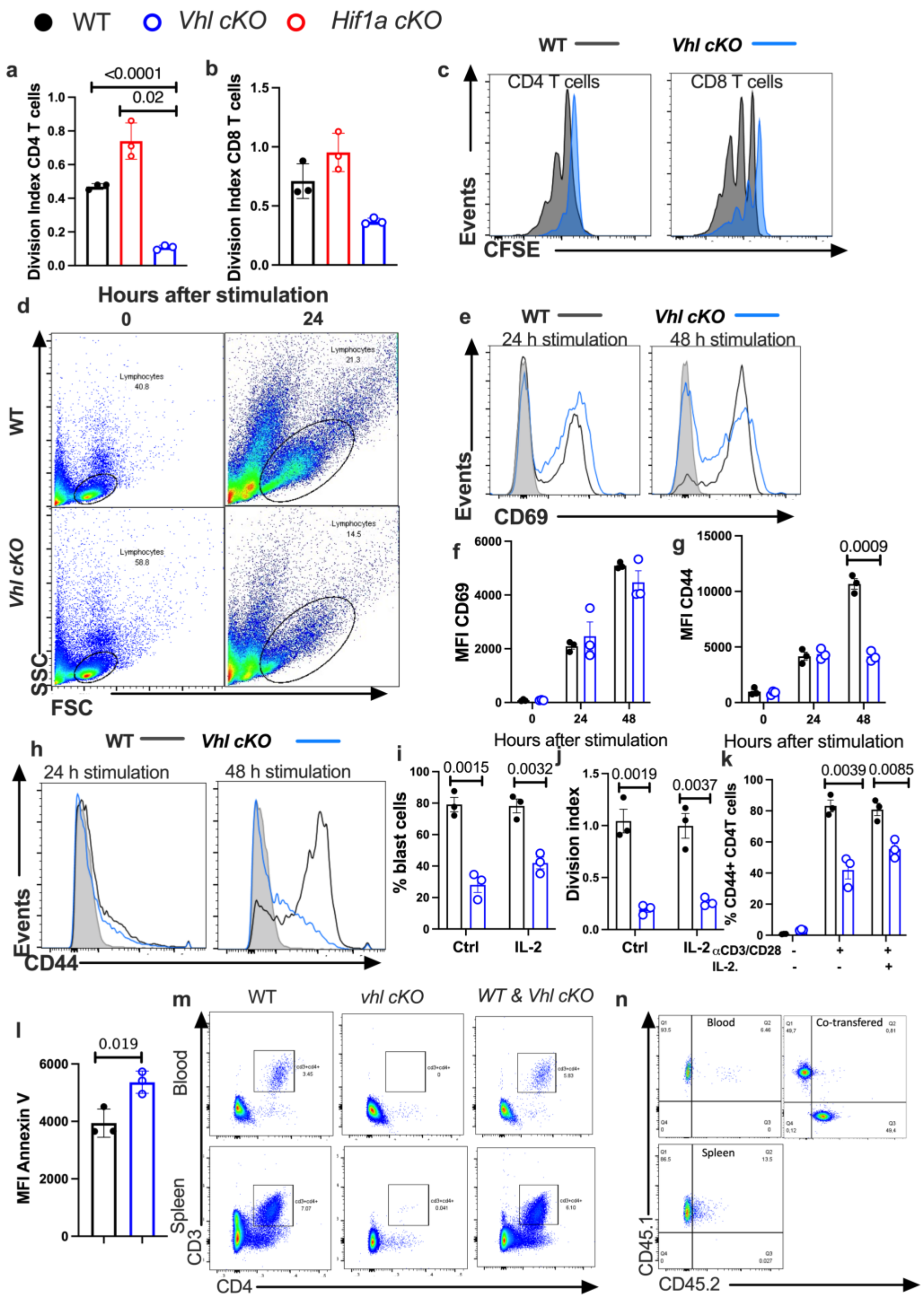

(a-c) The division index (the average number of cell divisions) (a, b) and a representative CFSE histogram profile (c) of CFSE labelled CD4 and CD8 T cells 3 days after stimulation with anti-CD3/ CD28 of WT, *Hif1a cKO* or *Vhl cKO* spleen cell suspensions is shown (n=3 independent cultures per group). (d) Representative dot plot showing the FCS/ SSC of *Vhl cKO* and WT total spleen cells before and 24 h after stimulation with anti-CD3/ CD28. (e, f) Representative histograms (e) and the MFI of CD69 expression (f) after anti-CD3/ CD28 stimulation of *Vhl cKO* and WT CD4 T cells are depicted. (g, h) Representative histograms (h) and the MFI of CD44 expression (g) after anti-CD3/ CD28 stimulation of *Vhl cKO* and WT CD4 T are shown. (i-k) WT and *Vhl cKO* CD4 T cells were stimulated with anti-CD3/ CD28 in presence or absence of 20 ng/ ml IL-2. The mean percentage of blast cells (i), the division index calculated by CFSE dilution (j) and the percentage of cells expressing CD44 (k) were determined 3 days after TCR-stimulation. (l) The MFI of Annexin V labeled *Vhl cKO* and WT CD4 T cells, 3 days after stimulation with anti-CD3/ CD28. (m, n) Groups of *Rag2*$^{-/-}$ mice were transferred i.v. with either 2.10^{6} CD4 T cells from CD45.2 *Vhl cKO*, CD45.1 WT or a mixed 1:1 suspension from both genotypes (10^{6} cells/ each). The representative dot plots of CD4 T cells (m) and of CD45.1 and CD45.2 in CD4 T cells before being co-transferred or in the blood and spleens of mice 5 weeks after co-transfer (n) are shown. Each symbol represents one mouse, and the data are presented as the mean ± SEM. n=3 biological independent samples per group. p-values were calculated using a one-way ANOVA with Welch's correction (a, b), and a two tailed unpaired Student's t test with a Welch correction and adjusted with FDR for multiple comparisons when proper (f, g, i-l). Source data are provided as a Source Data file.

Supplementary Figure 6

HIF-1 stabilization mediates the susceptibility to *M. tuberculosis*-infection of *Vhl cKO* mice

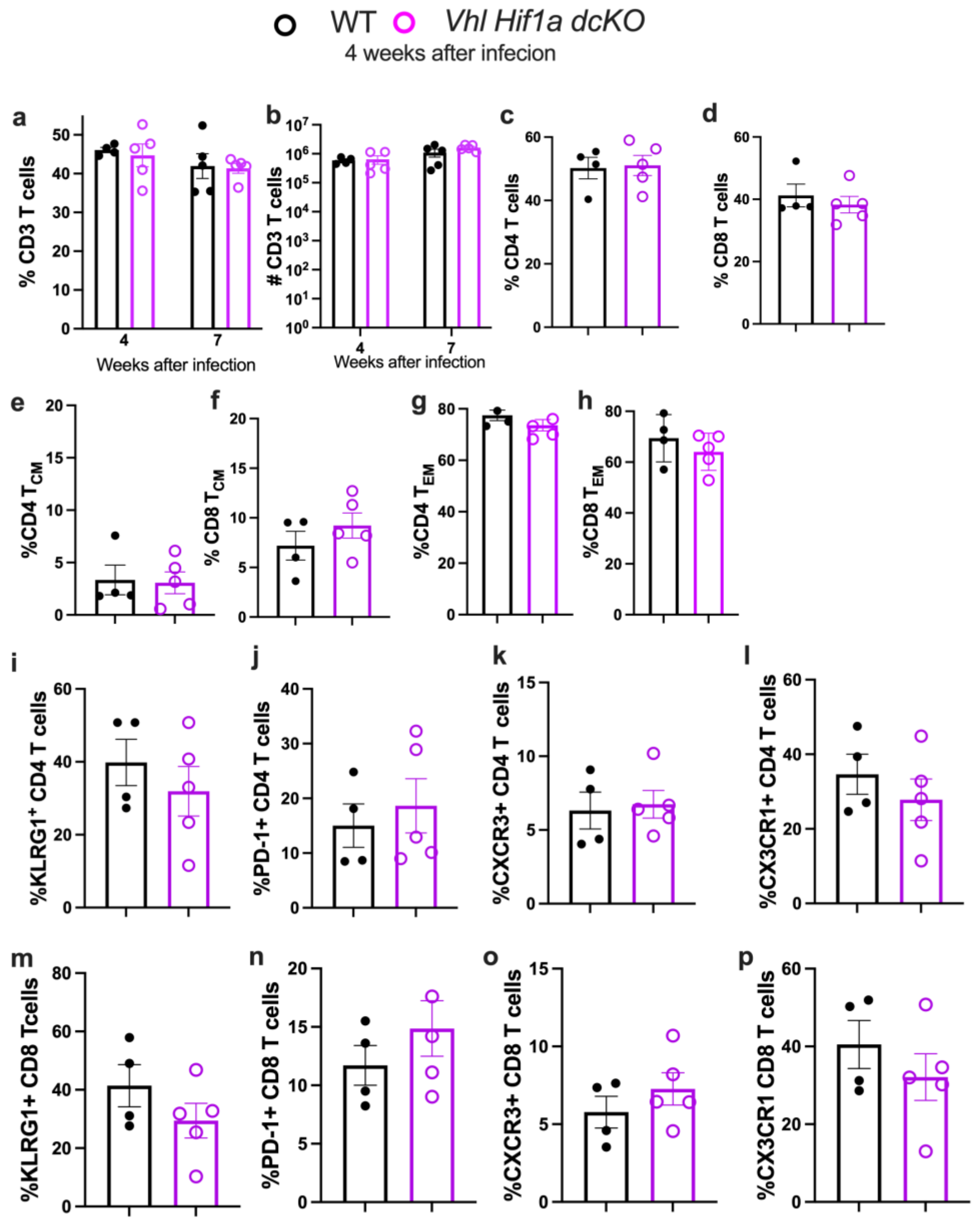

(a-d) The frequency (a) and number (b) of T cells, and the frequencies of CD4 (c) and CD8 (d) T cell populations in the lung of *Vhl Hif1a dcKO* and WT mice 4 weeks after *M. tuberculosis* infection are shown. (e-h) The frequencies of CD4 (e) and CD8 (f) T_{CM}, and of CD4 (g) and CD8

(h) T_{EM} in the lung of *Vhl Hif1a dcKO* and WT mice 4 weeks after *M. tuberculosis* infection are depicted. (i-p) The frequency of KLRG1+ (i, m), PD-1+ (j, n), CXCR3+ (k, o) and CX3CR1+ (l, p) CD4 (i-l) and CD8 (m-p) T cells in the lung of *Vhl Hif1a dcKO* and WT mice 4 weeks after infection with *M. tuberculosis* are shown. Each symbol represents one mouse, and the data are presented as the mean ± SEM; n=5 per group at 7 w.p.i; (a, b) WT n=4 *vhl hif1a dcKO* n=7 at 7 w.p.i respectively. p-values were calculated using a two tailed unpaired Student's t test with a Welch correction. Source data are provided as a Source Data file.

Supplementary Figure 7

HIF-1 stabilization impairs the spleen T cell responses to BCG immunization

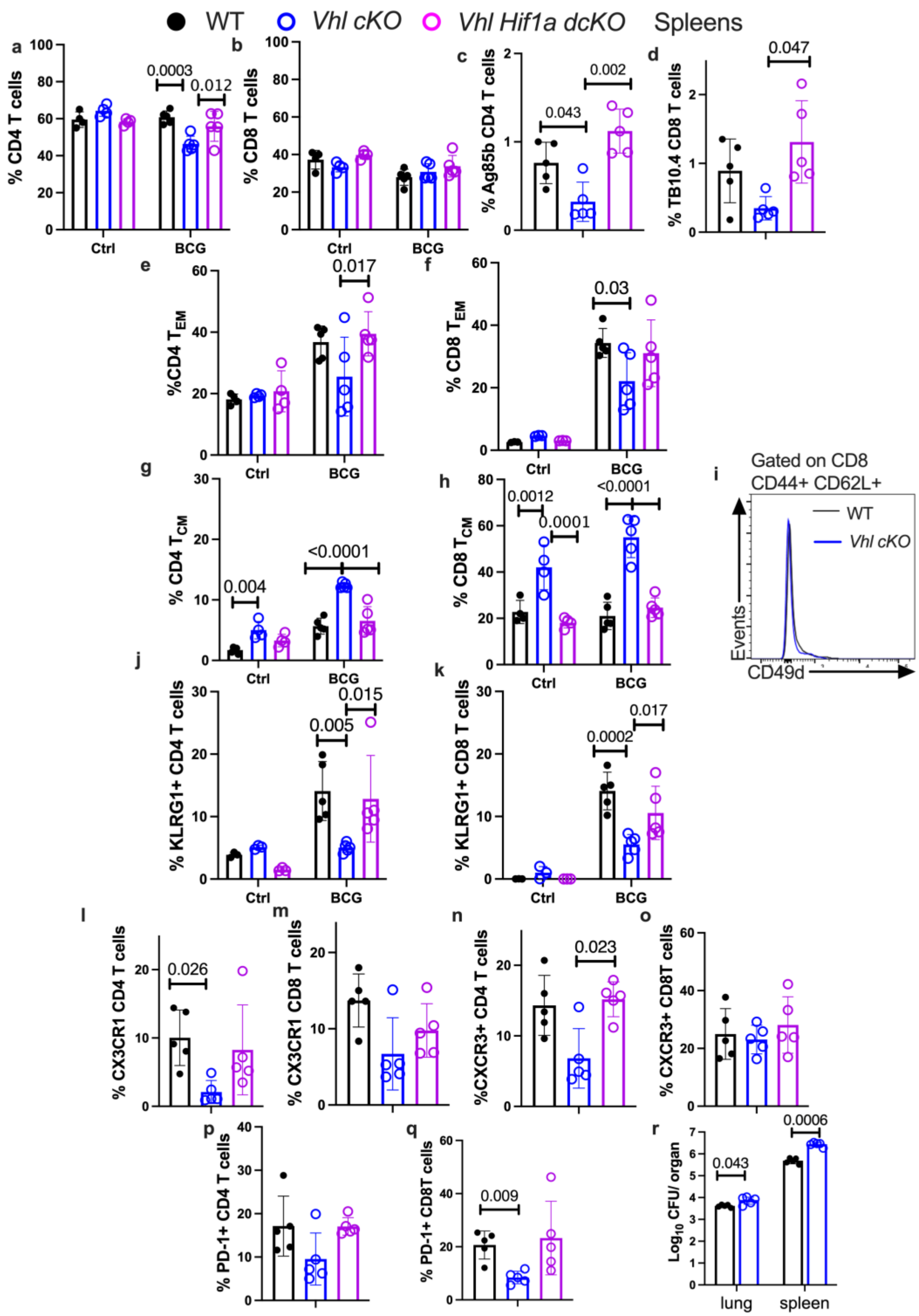

(a-d) The frequencies of total CD4 (a) and CD8 (b) T cells and of tetramer- Ag85b (c) and TB10.4-binding (d) CD4 and CD8 T cells respectively in the spleen from WT, *Vhl cKO* and *Vhl Hif1a dcKO* mice before and 3 weeks after i.v. immunization with 10^7 BCG are depicted. (e-h) The frequencies of CD4 T_{EM} (e), CD8 T_{EM} (f), CD4 T_{CM} (g) and CD8 T_{CM} (h) in the spleens from WT and mutant mice before and after BCG immunization. (i) Representative histogram of the expression of CD49d in spleen cells from *Vhl cKO* and WT non-immunized mice is shown. (j, k) The frequencies of KLRG1+ CD4 and CD8 T cells in the spleens of WT and mutant mice before and after BCG immunization is depicted. (l-q) The frequencies of CX3CR1+ (l, m), CXCR3+ (n, o) and PD-1+ (p, q) CD4 and CD8 T cells in lungs from *Vhl Hif1a dcKO*, *Vhl cKO* and WT mice 3 weeks after BCG immunization are depicted. (r) The $\log_{10}$ BCG CFU in the lungs and spleens of *Vhl cKO* and WT mice mice at 3 weeks after BCG immunization are depicted. Each symbol represents one mouse, and the data are presented as the mean ± SEM. WT n=4, 5; *Vhl cKO* n=5, 5 and *vhl hif1a dcKO* n=5, 5 at 0 and 3 weeks after immunization, respectively. p-values were calculated using 2-way ANOVA with Sidak adjustment (a, b, e-k) one way ANOVA with Welch correction (c, d, l-q) and a two-tailed unpaired t test with multiple with Welch correction and FDR adjustment for multiple comparison (r). Source data are provided as a Source Data file.

Supplementary Figure 8

HIF-1 stabilization impairs CD4 T cell responses in vitro

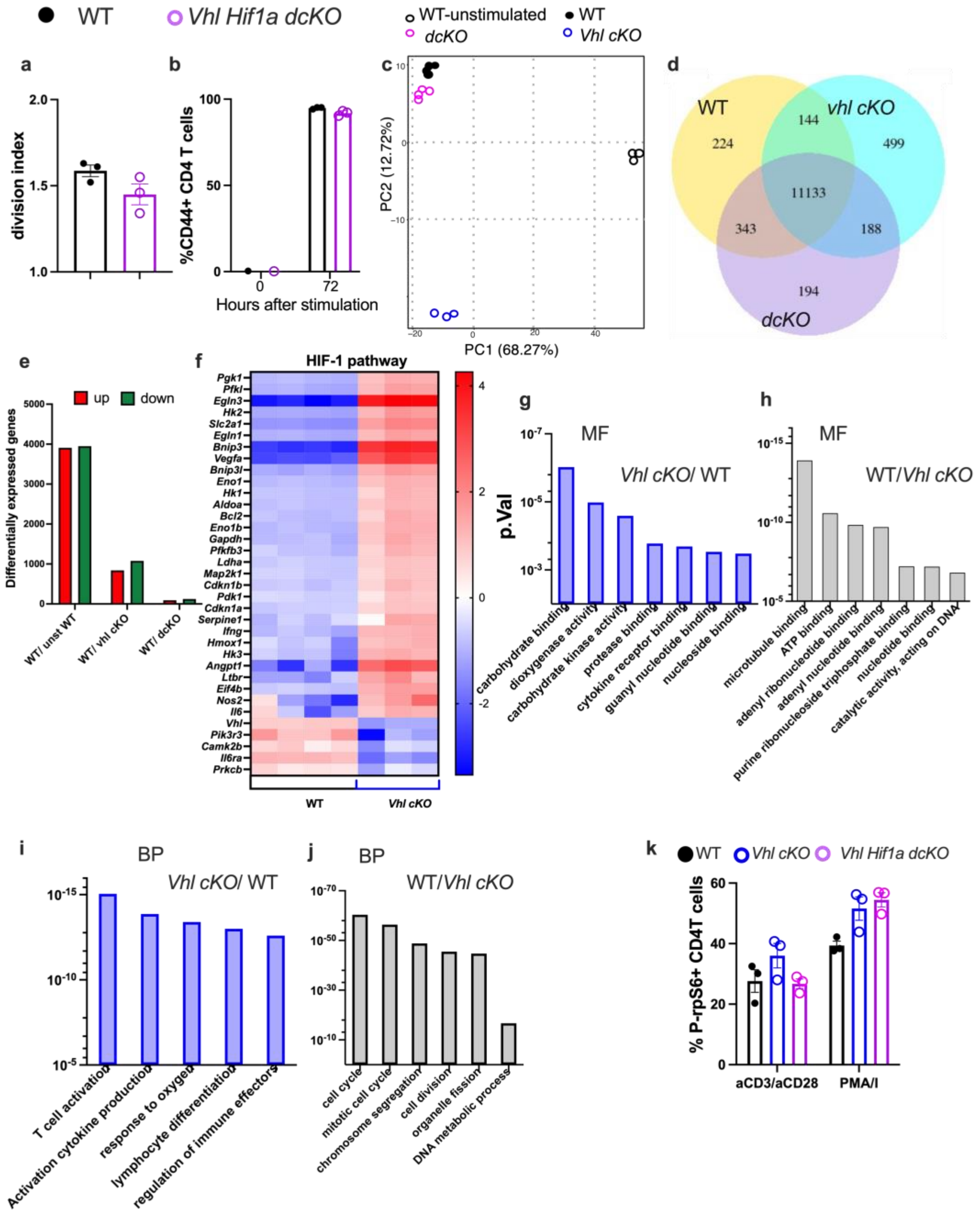

(a) The division index in CFSE labelled WT and *Vhl Hif1a dcKO* CD4 T cells 3 days after anti-CD3/CD28 stimulation is depicted. (b) The fraction of CD44+ WT and *Vhl Hif1a dcKO* CD4 T cells after TCR-stimulation. (c) Principal component analysis plots from non-supervised samples based on the normalized gene counts after filtering the low expressed genes are shown. (d) The Venn diagram represent the number of genes that are common to or uniquely expressed within WT, *Vhl cKO* and *Vhl Hif1a dcKO* CD4 T cells. (e) The number of differentially expressed genes (up or down-regulated) for each comparison combinations is shown. (f) The heat map of RNA-seq data showing HIF-1 pathway-specific genes differentially expressed in WT vs *Vhl cKO* CD4 T cells stimulated with anti-CD3/ CD28. Data were normalized by subtracting the $\log_2$ transformed values to the mean for each gene. (g-j) The $\log_{10}$ p-value for the most enriched terms after GO molecular function (MF) (g, h) and GO biological process (i, j) category analysis, in *Vhl cKO* vs WT (g, i) or in WT vs *Vhl cKO* CD4 T cells (h, j). (k) The frequency of phospho-rpSP6+ cells in WT and mutant CD4T cells measured 2 h after either PMA/ I or anti-CD3/ CD28 stimulation are shown. (a, b, k) Each symbol represents an independent biological sample, and the data are presented as the mean ± SEM, n= 3 samples per group. p-values were calculated using a two-tailed unpaired t test with multiple with Welch correction and (k) a 2-way ANOVA with Sidak adjustment. Source data are provided as a Source Data file.

Supplementary Figure 9

VHL expression allows TCR-mediated activation of CD4 T cells

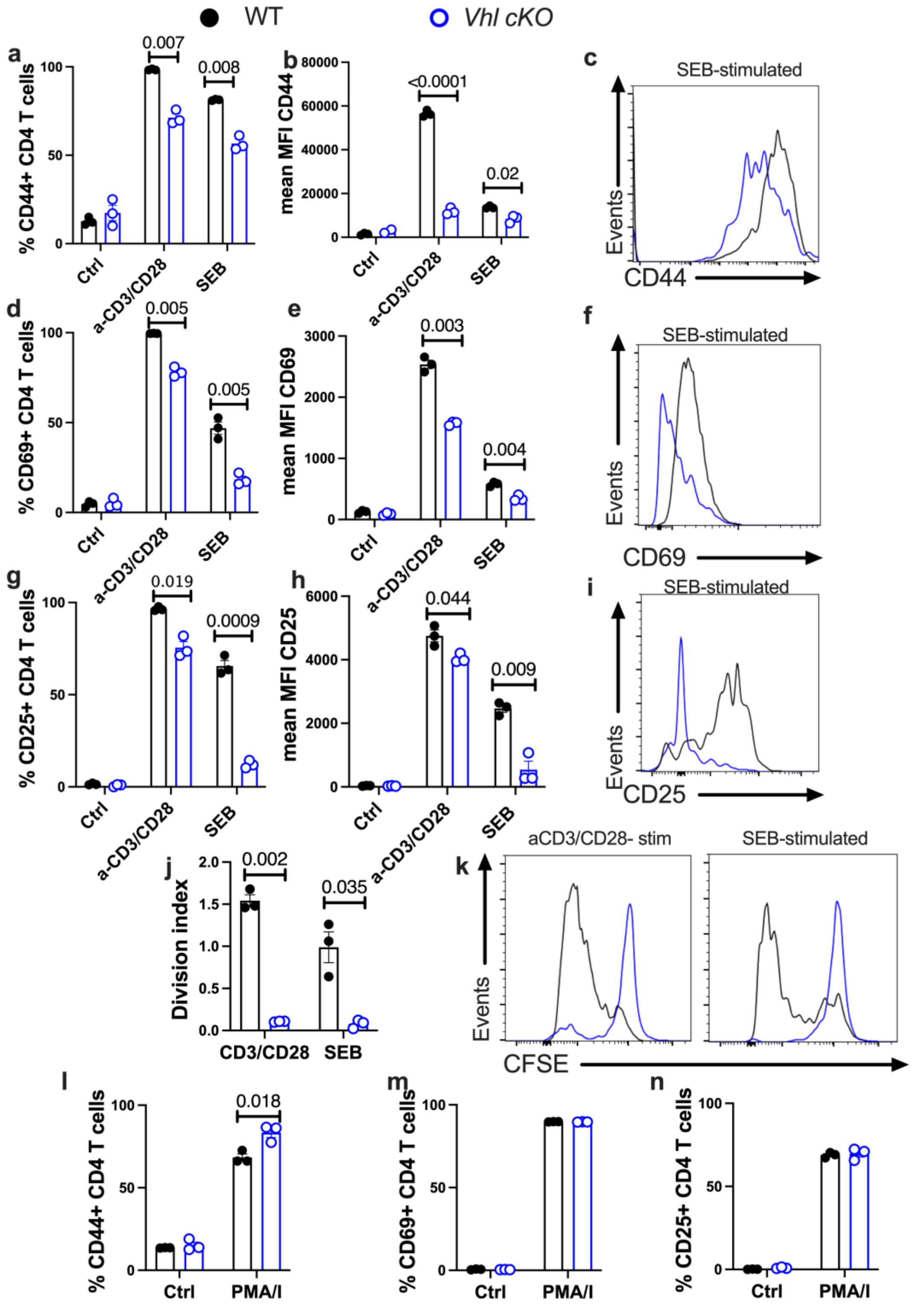

(a-i) The percentage of CD44, CD69 and CD25 positive cells (a, d, g) and the level of expression of these markers (b, e, h) were determined in *Vhl cKO* and WT CD4 T cells 72 h after incubation with either anti-CD3/ CD28, SEB or control medium. (c, f, i) Representative histograms of CD44, CD69 and CD25 expression on WT and *Vhl cKO* CD4 T cells 72 h after SEB-stimulation are shown. (j, k) Representative profiles (k) of CFSE-labelled *Vhl cKO* and WT CD4 T cells 3 days after stimulation with anti-CD3/ CD28 or SEB and the mean division index ± SEM (j) are shown. (l-n) The percentage of CD44 (l), CD69 (m) and CD25 (n) expressing WT and *Vhl cKO* CD4 T cells were determined 24h after PMA/ Ionomycin stimulation. Each symbol represents an independent biological sample, and the data are presented as the mean ± SEM; n= 3 samples per group. p-values were calculated using a two-tailed unpaired *t*-test with Welch's correction and FDR adjustment for multiple comparisons. Source data are provided as a Source Data file.

Supplementary figure 10

Flow cytometry gating strategy for ex vivo and in vitro studies

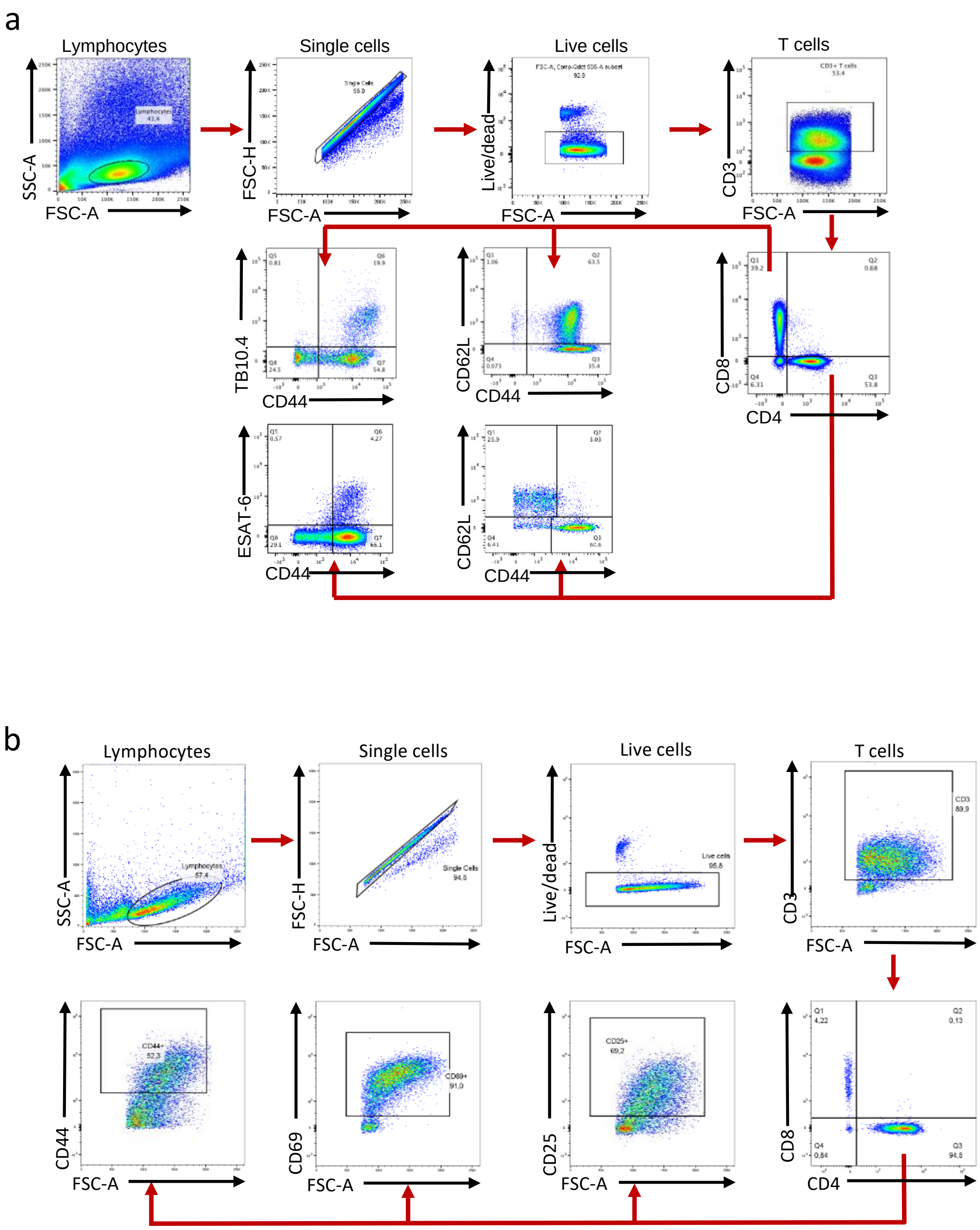

a) Example of the flow cytometry gating strategy for the analysis of T cells from the lung of WT mice 8 weeks after aerosol infection with *M. tuberculosis*.

b) Example of the flow cytometry gating strategy for the analysis of spleen CD4T cells, 72h after anti-CD3/ CD28 activation.

Supplementary Table 1

Highest ranked differentially expressed genes in Vhl cKO / WT CD4 T cells after TCR stimulation.

Order	Gene name	Gene symbol	Involved in
1	phosphoglycerate kinase 1	Pgk1	Glycolysis/ gluconeogenesis
2	triosephosphate isomerase 1	Tpi1	Glycolysis/ gluconeogenesis
3	phosphofructokinase, liver, B-type	Pfkp	Glycolysis/ gluconeogenesis
4	phosphofructokinase, liver, B-type. Liver	Pfkl	Glycolysis/ gluconeogenesis
5	egl-9 family hypoxia-inducible factor 3	Egln3	Oxygen sensing
6	solute carrier family 2 member 1	Slc2a3	Transporter
7	interferon inducible GTPase 1	Iigp1	Immune response
8	solute carrier family 16 (member 3	Slc16a3	Transporter
9	hexokinase 2	Hk2	Glycolysis/ gluconeogenesis
10	solute carrier family 2 member 1	Slc2a1	Transporter
11	egl-9 family hypoxia-inducible factor 1	Egln1	Oxygen sensing
12	integrin alpha 7	Itga7	Immune response
13	lymphocyte antigen 6 complex, locus C1	Ly6c1	Immune response
14	granzyme B	Gzmb	Immune response
15	annexin A2	Anxa2	Angiogenesis
16	GLI pathogenesis-related 2	Glipr2	
17	aldo-keto reductase family 1, member C18	Akr1c18	
18	BCL2/adenovirus E1B interacting protein 3]	Bnip3	Mitochondrial responses
19	phosphoglucomutase 2	Pgm2	Glycolysis/ gluconeogenesis
20	adenylate kinase 4	Ak4	Mitochondrial responses
21	N-myc downstream regulated gene 1	Ndrg1	Inhibition proliferation
22	vascular endothelial growth factor A	Vegfa	Angiogenesis
23	activating transcription factor 3	Atf3	Angiogenesis
24	regulator of G-protein signaling 11	Rgs11	
25	aldolase C, fructose-bisphosphate	Aldoc	Glycolysis/ gluconeogenesis
26	lectin, galactose binding, soluble 3	Lgals3	
27	interleukin 10 receptor, alpha	Il10ra	Immune response
28	pyruvate kinase, muscle	Pkm	Glycolysis/ gluconeogenesis
29	BCL2/adenovirus E1B interacting protein 3-like	Bnip3l	Mitochondrial responses
30	SLAM family member 7	Slamf7	Immune response
31	ERO1-like (S. cerevisiae)	Ero1l	
32	procollagen-proline, 2-oxoglutarate 4-dioxygenase alpha 1 polypeptide	P4ha1	Oxygen sensing
33	aldo-keto reductase family 1, member C12	Akr1c12	
34	MAX interactor 1, dimerization protein	Mxi1	Inhibition proliferation
35	protein phosphatase 2, regulatory subunit B'', alpha	Ppp2r3a	Inhibition proliferation
36	interleukin 2 receptor, alpha chain	Il2ra	Immune response
37	beta galactoside alpha 2,6 sialyltransferase 1	St6gal1	
38	enolase 1, alpha non-neuron	Eno1	Glycolysis/ gluconeogenesis
39	procollagen lysine, 2-oxoglutarate 5-dioxygenase 2	Plod2	Oxygen sensing
40	Kruppel-like factor 6	Klf6	Inhibition proliferation
41	glycogen synthase 1, muscle	Gys1	Glycolysis/ gluconeogenesis
42	polypeptide N-acetylgalactosaminyltransferase 6	Galnt6	
43	histocompatibility 2, class II antigen A, beta 1	H2-Ab1	Immune response
44	hexokinase 1	Hk1	Glycolysis/ gluconeogenesis
45	procollagen-proline, 2-oxoglutarate 4-dioxygenase, alpha II polypeptide	P4ha2	Oxygen sensing

Supplementary table 2

**Primary Abs for Flow cytometry.**	**Fluorochrome**	**Clone**	**Company**	**Cat Nr**	
CD16/CD32		2.4G2	BD	553142	
CD3e	eFlour450	17A2	eBioscience™	48-0032-82	
CD4	BV786	GK1.5	BD	563331	
CD8a	Alexa Fluor 700	3B5	eBioscience™	56-0081-82	
CD44	BV711	OX-49	eBioscience	67-0441-82	
CD69	APC-Cyanine7	FN50	eBioscience™	47-0691-82	
CD25	V450	PC61	BD	561257	
CD62L	BV711a	MEL14	BioLegend	104445	
CD71	FITC	C2	BD	561936	
CXCR3	PE/Dazzle 594	CXCR3-173	Biolgend	126533	
CX3CR1	PE-Cyanine7	SA011F11	BioLegend	149016	
PD-1	BV605	29F.1A12	BioLegend	135219	
KLRG1	BV650	2F1	BD	740553	
TCR-β	FITC	H57-597	eBioscience™	11-5961-82	
TCRγδ	APC	eBioGL3	eBioscience™	17-5711-81	
CD3	-	145-2C11	Biolgend	100302	
CD28	-	37,51	BD Pharmingen	553295	
CD49d		9C10	Biolegend	304309	
IFN-γ	APC	XMG1.2	eBioscience™	17-7311-82	
FOXP3	APC	FJK-16s	Invitrogen	17-5773-82	
Ki-67	PE	SolA15	Invitrogen	12-5698-82	
CTLA4	PE	UC10-4F10-11	BD	564332	
CD45.2	BV711	104	Biolegend	109847	
CD45.2	FITC	104	Biolegend	109805	
CD45.1	V450	A20	BD	560520	
CD49d	PE	R1-2	BD	564395	
Phospho-S6 (Ser235, Ser236)	PE	cupk43k	Invitrogen	12-9007-42	
**Primary antibodies for WB (Ag)**					
c-Myc	-	Y69	Abcam	ab32072	
actin	-	A5441	Sigma	A5441	
HIF-1a	-	polyclonal	Novus Biologicals	NB-100-479	
GAPDH	-	polyclonal	Abcam	ab22555	
**Secondary antibodies**					**Dilutions**
anti-rabbit HRP-conjugated	-	polyclonal	Abcam	ab97080	1:10000
anti-mouse HRP-conjugated	-	polyclonal	Abcam	ab97046	1:5000
Peroxidase AffiniPure Goat Anti-Mouse IgG (H+L)		polyclonal	Jackson ImmunoResearch	115-035-166	1:5000

Supplementary table 3

List and sequences of RT-PCR primers used

Primer	Forward	Reverse
vegfa	TAGAGTACATCTTCAAGCCG	TCTTTCTTTGGTCTGCATTC
ldha	TGGCAGACTTGGCTGACAG	ACCTTCACAACATCCGAGATTC
mxi1	ACCAGCATCAGTGACCTTGA	CTAGGACGCGAAGGAGAGTT
cd71	GCGCTTCCTAGTACTCCCTT	CTGCAGCCAGTTTCATCTCC
cmyc	CCACCAGCAGCGACTCTG	GAGATGAGCCCGACTCCG
cdkn1a	CAACCCATCTGCATCCGTTT	TGGCCTTAGAGGTGACAAGG
cdkn1b	CCCGAGGAGGAAGATGTCAA	CTCCAAGTCCCGGGTTAGTT
hprt	CCCAGCGTCGTGATTAGC	GGAATAAACACTTTTTCCAAATCC
pdk1	GAAGCAGTTCCTGGACTTCG	CCAACTTTGCACCAGCTGTA
