# Supplementary material for: HIF-1 stabilization in T cells hampers the control of Mycobacterium tuberculosis infection
Source: Nat Commun. 2022 Sep 5;13:5093. doi: 10.1038/s41467-022-32639-9 (PMC9445005; doi:10.1038/s41467-022-32639-9)
Supplement: Supplementary file 3 — Reporting Summary [file 41467_2022_32639_MOESM3_ESM.pdf]

nature portfolio

Corresponding author(s): Martin Rottenberg

Last updated by author(s): Jul 23, 2022

Reporting Summary

Nature Portfolio wishes to improve the reproducibility of the work that we publish. This form provides structure for consistency and transparency in reporting. For further information on Nature Portfolio policies, see our Editorial Policies and the Editorial Policy Checklist.

Statistics

For all statistical analyses, confirm that the following items are present in the figure legend, table legend, main text, or Methods section.

n/a	Confirmed	
☐	☒	The exact sample size (*n*) for each experimental group/condition, given as a discrete number and unit of measurement
☐	☒	A statement on whether measurements were taken from distinct samples or whether the same sample was measured repeatedly
☐	☒	The statistical test(s) used AND whether they are one- or two-sided<br>*Only common tests should be described solely by name; describe more complex techniques in the Methods section.*
☒	☐	A description of all covariates tested
☐	☒	A description of any assumptions or corrections, such as tests of normality and adjustment for multiple comparisons
☐	☒	A full description of the statistical parameters including central tendency (e.g. means) or other basic estimates (e.g. regression coefficient) AND variation (e.g. standard deviation) or associated estimates of uncertainty (e.g. confidence intervals)
☐	☒	For null hypothesis testing, the test statistic (e.g. *F*, *t*, *r*) with confidence intervals, effect sizes, degrees of freedom and *P* value noted<br>*Give P values as exact values whenever suitable.*
☒	☐	For Bayesian analysis, information on the choice of priors and Markov chain Monte Carlo settings
☒	☐	For hierarchical and complex designs, identification of the appropriate level for tests and full reporting of outcomes
☒	☐	Estimates of effect sizes (e.g. Cohen's *d*, Pearson's *r*), indicating how they were calculated

Our web collection on statistics for biologists contains articles on many of the points above.

Software and code

Policy information about availability of computer code

Data collection	FACSDiva 6.0.
Data analysis	GraphPad Prism 9 software version 3; Excel; FlowJow v10.7

For manuscripts utilizing custom algorithms or software that are central to the research but not yet described in published literature, software must be made available to editors and reviewers. We strongly encourage code deposition in a community repository (e.g. GitHub). See the Nature Portfolio guidelines for submitting code & software for further information.

Data

Policy information about availability of data

All manuscripts must include a data availability statement. This statement should provide the following information, where applicable:

- Accession codes, unique identifiers, or web links for publicly available datasets
- A description of any restrictions on data availability
- For clinical datasets or third party data, please ensure that the statement adheres to our policy

The raw and processed RNA sequencing data have been deposited in the publicly accessible Gene Expression Ommnibus (GEO) NIH database repository and can be accessed using GEO accession numbers GSE190791 (https://www.ncbi.nlm.nih.gov/geo/query/acc.cgi?acc=GSE190791) and GSE190909 (https://www.ncbi.nlm.nih.gov/geo/query/acc.cgi?acc=GSE190909). The authors declare that data supporting the findings of this study are available within the paper and its supplementary information files. A Source data are provided with this paper.

March 2021

Human research participants

Policy information about [studies involving human research participants and Sex and Gender in Research.]

Reporting on sex and gender	N/A
Population characteristics	N/A
Recruitment	N/A
Ethics oversight	N/A

Note that full information on the approval of the study protocol must also be provided in the manuscript.

Field-specific reporting

Please select the one below that is the best fit for your research. If you are not sure, read the appropriate sections before making your selection.

☒ Life sciences ☐ Behavioural & social sciences ☐ Ecological, evolutionary & environmental sciences

For a reference copy of the document with all sections, see nature.com/documents/nr-reporting-summary-flat.pdf

Life sciences study design

All studies must disclose on these points even when the disclosure is negative.

Sample size	Sample size were both indicated in the legend captions manuscript or the parametric results for all individuals for each sample plotted. The sample size was based based on our previous scientific experience and on standards in the field, allowing us to make inferences from the population from the sample (see for example Nature Communications volume 13, Article number: 884 (2022)) . Studies in vivo in which the bacterial load were determined were done using more than 6-11 individuals per sample , while when determining cellular immune parameters (T cell subpopulations, their activation status, differentiation or function) used 4-8 individuals per sample. When performing in vitro experiments 3 independent biological replicates at least were included per experiment.
Data exclusions	No data were excluded from the analysis.
Replication	We have replicated all our determinations in independent experiments in the study. Some of the in vivo studies in which different time points after infection were analysed were not replicated if observations at different times after infection (i.e. Fig 1 a, b that comprise 60 animals). Although not a proper repetition Fig 7a, b shown the same infomation (increased CFU in lungs and spleens of Vhl cKO compared to WT mice). Otherwise the experiments were repeated at least twice.
Randomization	The mutant mouse strains used were bred as cre +/- heterozygotes and genotyped before used for in vivo or in vitro studies. Experiments were done comparing littermates. The samples were otherwise allocated randomly to each group. If possible sex matched individuals were used.
Blinding	Blinding was not relevant to our study because data are obtained with unbiased quantitative measurements (not observation determined) . The genotype of the organs was blinded to the investigator performing the CFU determinations.

Reporting for specific materials, systems and methods

We require information from authors about some types of materials, experimental systems and methods used in many studies. Here, indicate whether each material, system or method listed is relevant to your study. If you are not sure if a list item applies to your research, read the appropriate section before selecting a response.

Materials & experimental systems

n/a	Involved in the study	
☐	☒	Antibodies
☒	☐	Eukaryotic cell lines
☒	☐	Palaeontology and archaeology
☐	☒	Animals and other organisms
☒	☐	Clinical data
☒	☐	Dual use research of concern

Methods

n/a	Involved in the study	
☒	☐	ChIP-seq
☐	☒	Flow cytometry
☒	☐	MRI-based neuroimaging

Antibodies

Antibodies used

Fluorochrome labelled Abs.	Fluorochrome	Clone	Company	Cat Nr
CD16/CD32		2.4G2	BD	553142
CD3e	eFlour450	17A2	eBioscience™	48-0032-82
CD4	BV786	GK1.5	BD	563331
CD8a	Alexa Fluor 700	3B5	eBioscience™	56-0081-82
CD44	BV711	OX-49	eBioscience	67-0441-82
CD69	APC-Cyanine7	FN50	eBioscience™	47-0691-82
CD25	V450	PC61	BD	561257
CD62L	BV711a	MEL14	BioLegend	104445
CD71	FITC	C2	BD	561936
CXCR3	PE-Dazzle 594	CXCR3-173	Biolegend	126533
CX3CR1	PE-Cyanine7	SA011F11	BioLegend	149016
PD-1	BV605	29F.1A12	BioLegend	135219
KLRG1	BV650	2F1	BD	740553
TCR-β	FITC	H57-597	eBioscience™	11-5961-82
TCRγδ	APC	eBioGL3	eBioscience™	17-5711-81
CD3		145-2C11	Biolegend	100302
CD28		37,51	BD	553295
CD49d		9C10	Biolegend	304309
IFN-γ	APC	XMG1.2	eBioscience™	17-7311-82
FOXP3	APC	FJK-16s	Invitrogen	17-5773-82
Ki-67	PE	SolA15	Invitrogen	12-5698-82
CTLA4	PE	UC10-4F10-11	BD	564332
CD45.2	BV711	104	Biolegend	109847
CD45.2	FITC	104	Biolegend	109805
CD45.1	V450	A20	BD	560520
CD49d	PE	R1-2	BD	564395
Phospho-S6 (Ser235,236)	PE	cupk43k	Invitrogen	12-9007-42

Primary antibodies for WB.

c-Myc -	Y69	Abcam	ab32072
actin	A5441	Sigma	A5441
HIF-1a -	polyclonal	Novus Biologicals	NB-100-479
GAPDH -	polyclonal	Abcam	ab22555

Secondary antibodies
anti-rabbit HRP-conjugated - polyclonal Abcam ab97080 dil 1:10000
anti-mouse HRP-conjugated - polyclonal Abcam ab97046 dil 1:5000
Peroxidase AffiniPure Goat Anti-Mouse IgG (H+L) polyclonal Jackson ImmunoResearch 115-035-166 dil 1:5000

Validation

The validation of all primary commercial antibodies for the species and application was warranted by the vendors. Validation statement can be found on the manufacturers' website. We include one reference for each primary antibody used.

CD16/CD32 https://pubmed.ncbi.nlm.nih.gov/8406898
CD3e http://www.ncbi.nlm.nih.gov/pubmed/32733463
CD4 https://pubmed.ncbi.nlm.nih.gov/10562325
CD8a http://www.ncbi.nlm.nih.gov/pubmed/32328060
CD44 http://www.ncbi.nlm.nih.gov/pubmed/33933782
CD69 http://www.ncbi.nlm.nih.gov/pubmed/33731208
CD25 https://pubmed.ncbi.nlm.nih.gov/3919312
CD62L https://www.ncbi.nlm.nih.gov/pubmed/30540933
CD71 https://pubmed.ncbi.nlm.nih.gov/2526689
CXCR3 https://pubmed.ncbi.nlm.nih.gov/33271118
CX3CR1 https://www.ncbi.nlm.nih.gov/pubmed/29504946
PD-1 https://www.ncbi.nlm.nih.gov/pubmed/29249639
KLRG1 https://pubmed.ncbi.nlm.nih.gov/11745363
TCR-β http://www.ncbi.nlm.nih.gov/pubmed/32966787
TCRγδ http://www.ncbi.nlm.nih.gov/pubmed/33088477
CD3 https://www.ncbi.nlm.nih.gov/pubmed/29184112
CD28 https://pubmed.ncbi.nlm.nih.gov/7540940
CD49d https://www.ncbi.nlm.nih.gov/pubmed/29359991
IFN-γ http://www.ncbi.nlm.nih.gov/pubmed/27390762
FOXP3 http://www.ncbi.nlm.nih.gov/pubmed/29021893
Ki-67 http://www.ncbi.nlm.nih.gov/pubmed/34151227
CTLA4 https://pubmed.ncbi.nlm.nih.gov/8943377
CD45.2 https://www.ncbi.nlm.nih.gov/pubmed/28709804
CD45.2 https://www.ncbi.nlm.nih.gov/pubmed/28542148
CD45.1 https://pubmed.ncbi.nlm.nih.gov/2466938
CD49d https://pubmed.ncbi.nlm.nih.gov/7523506
Phospho-S6 (Ser235, Ser236) http://www.ncbi.nlm.nih.gov/pubmed/29920188

anti-c-Myc (website) https://www.abcam.com/hrp-c-myc-antibody-y69-ab205818.html
anti-actin https://pubmed.ncbi.nlm.nih.gov/19756912/
anti-HIF-1a http://www.ncbi.nlm.nih.gov/pubmed/34997404
anti-GAPDH https://www.ncbi.nlm.nih.gov/pubmed/33181214?dopt=Abstract

Animals and other research organisms

Policy information about studies involving animals; ARRIVE guidelines recommended for reporting animal research, and Sex and Gender in Research

Laboratory animals	All mice in this study were between 8-15 week/ old. Mice were maintained in a specific pathogen free unit on a 12hr light/12hr dark cycle. Room temperature was maintained at 25°C. The humidity level was controlled between 40-60%. Mice containing loxP-flanked Hif1a and Vhl alleles were provided by Dr Randall Johnson and crossed to cd4 cre or dlck cre transgenic mice. Rag2 KO and C57Bl/6 mice were purchased from Janvier labs. The Ly5.1 B6 congenic strain was provided by Dr Benedict Chambers.
Wild animals	This study did not involve wild animals
Reporting on sex	Not relevant: Tuberculosis susceptibility is not gender related. Both females and male mice were used.
Field-collected samples	This study did not involve samples collected from the field.
Ethics oversight	The studies were approved by the Stockholm North Region Animal Research Ethical Committee and were treated following local, Swedish national and EU guidelines.

Note that full information on the approval of the study protocol must also be provided in the manuscript.

Flow Cytometry

Plots

Confirm that:

☒ The axis labels state the marker and fluorochrome used (e.g. CD4-FITC).

☒ The axis scales are clearly visible. Include numbers along axes only for bottom left plot of group (a 'group' is an analysis of identical markers).

☒ All plots are contour plots with outliers or pseudocolor plots.

☒ A numerical value for number of cells or percentage (with statistics) is provided.

Methodology

Sample preparation	Lungs were removed, mechanically minced into small pieces and digested with 3 mg/ ml Collagenase D and 30 ug/ ml DNase I for 1 h at 37oC, and single-cell suspensions prepared by filtering lung tissue through 70 µm nylon cell strainers. To enrich the suspension in lymphocytes, cells were loaded into an isotonic 40-70% Percoll density gradient and centrifuged for 30 min at room temperature. Cells in the gradient interphase were collected and washed before further culturing/ labelling. Mediastinal lymph node cell suspensions were obtained after mechanical disruption of the followed by filtering over a 70-µm nylon mesh. Single spleen cell suspensions were obtained by mechanical disruption, lysis of erythrocytes and straining over a 70-µm nylon mesh. Cell suspensions were incubated with live/dead stain (LIVE/DEAD™ Fixable Yellow Dead Cell Stain, Invitrogen). Then CD16/CD32 blocking antibodies (BD) and the fluorophore conjugated antibody cocktails were introduced and incubated for 30 minutes on ice. Cells were then washed with PBS, resuspended and fixed with 2% paraformaldehyde solution in PBS.<br><br>For isolation of naive T cells and in vitro activation: Single cell suspensions were obtained from spleens of mice by mechanical disruption, filtering over a 70 um nylon cell strainer and lysis of erythrocytes. Label-free naïve CD4+CD44- T cells were further isolated by negative selection using magnetic beads (MACS, Miltenyi Biotec, Germany). After counting, splenocytes were resuspended in PBS with 0.5% bovine serum albumin (BSA) and 2 mM EDTA, incubated with biotin-conjugated anti-CD44 microbeads and applied through a separation column under a magnetic field. Selected cells were then resuspended in RPMI-1640 media supplemented with 5% FCS and penicillin/streptomycin stimulated in vitro with either plate-bound anti-CD3 antibody (145-2C11, Invitrogen, Waltham, MS) and soluble anti-CD28 antibody (37.51, BD, Franklin Lakes, NJ), with 50 ng/ml phorbol myristate acetate (PMA) and 2 ug/ml ionomycin (Sigma, StLouis, MO), or with 20 ug/ ml Staphylococcal enterotoxin B (SEB) (Sigma) and cultured at CO2 incubator at 37°C and 5% CO2.
Instrument	Data were acquired on a LSRII or a FACS Canto II flow cytometers using a BD Diva Software.
Software	Data were analyzed with FlowJo software (Tree star Inc., Ashland, OR). Raw numbers for each group were exported to excel. Following additional calculations, GraphPad Prism was used to plot and statistically analyze the percent and total number of the selected populations.
Cell population abundance	The number of single cells (identified as those cells with appropriate SSC-A, FSC-A and FSC-H parameters in Flow Jo, (as shown) was used to calculate the percent of population abundance for each cell population. Total number of cells for each subpopulation was then calculated using the percentage obtained as described and then multiplied by the calculated number of cells for each sample as counted under the microscope at a known dilution. We acquired 5-10.10 exp 5- lung cells for ex

vivo studies and 5-10. 10 exp 4 cells in studies in vitro.

Gating strategy

The SSC-A and FSC-A parameters were used to identify "lymphocyte populations" excluding cellular debris (bottom right corner). Following the identification of lymphocyte populations, doublets were removed, and single cells were selected based on FSC-A and FSC-H as indicated. The single cells as selected using FSC-A and FSC-H accounted for approximately 95% of live cells as determined using the "LIVE/DEAD" stain. This was followed by a CD3 gate, followed by a CD4 and CD8 gate. Subsequently CD44hi mycobacteria specific T cells were gated using MHCI or MHCII tetramers (or when indicated by their ability to release IFN-g after peptide stimulation). Tetrtamer + or total CD44h CD4 or CD8 T cells were used for the expression of activation/ differentiation or inhibitory markers (CD69, PD-1, CTLA-4, CXCR3, CX3CR1, CD62L, KLRG-1, CD49d, CD71, CD25). For in vitro studies total CD4 T cells were gated for further analysis. A figure exemplifying the gating strategy is provided with the Supplementary figures. For

☒ Tick this box to confirm that a figure exemplifying the gating strategy is provided in the Supplementary Information.
